# Generation, types and impacts of biomedical waste during COVID-19: Indian context

**DOI:** 10.5620/eaht.2023019

**Published:** 2023-10-10

**Authors:** Shefali

**Affiliations:** Maharshi Dayanand University, Rohtak, India

**Keywords:** Bio-Medical waste, COVID-19, Waste generation, Types of waste, Impacts, Management

## Abstract

India's already-stressed waste disposal system has been strained by the COVID-19 outbreak. The challenge of managing biomedical Waste (BMW) in India has become more complicated in recent years, mainly due to the sudden emergence of COVID-19. As a methodology, a literature review was carried out with the help of Google Scholar, Research Gate, PubMed, and academic journal databases. Additionally, publications from numerous non-academic sources (such as news websites etc.) and current guidelines, such as those from the Ministry of Health and Family Welfare, Government of India, were also accessed. The review study identifies that PPE (Personal Protective Equipment) made up the majority of biomedical waste, followed by vaccine waste, during the peak of the COVID-19 vaccination campaign. The increase in PPEs such as face masks, aprons, face shields, gloves, goggles, and sanitizers, as well as other medical waste like bandages, plastic containers, syringes, testing kits, and tissues, has significantly changed the makeup of the BMW. This pandemic has hampered the proper management of solid waste, which has now surfaced as a major threat to developing countries. In this paper, biomedical waste management strategies followed in India and its disposal methods, cost-effective and environment-friendly methods to treat medical waste are also discussed.

## Background

In December 2019, novel corona virus disease caused by the virus SARS CoV-2 (severe acute respiratory syndrome coronavirus 2), spread throughout Wuhan, China, and had symptoms similar to pneumonia [1]. On February 11, 2020, the WHO named the new illness "COVID-19” [2]. Within a short period of time, the disease began to spread outside of China, infecting the majority of continents and nations, and forming a pandemic. India reported its first COVID-19 case on January 30, 2020, in the state of Kerala with a history of travel to Wuhan [2]. The COVID-19 outbreak was declared a pandemic and a public health emergency by the World Health Organization on March 11, 2020. By causing significant changes in daily life, the corona virus outbreak has had a negative impact on both the individual and social elements of human beings [3]. Along with negatively affecting public health, the pandemic also had an impact on the socioeconomic standing of several nations. The impact was especially pronounced in South Asian nations like India because of their enormous populations, high rates of poverty, and inadequate healthcare infrastructure [4].

The corona virus pandemic has had a significant impact on municipal solid waste management (MSWM) and changed the type and amount of waste. The term "MSW" refers to an assortment of different sorts of solid waste created by residents of a community, whether in an urban or rural setting, where there is a possibility of direct physical contact between people [5]. In situations where the infected or suspected person is being treated or cared for at home, touching contaminated surfaces and objects during household waste management and the potential for produced aerosol during waste handling are possible ways of transmitting infectious diseases such as the corona virus through household waste [3]. It has been noted that the causal virus can survive on various surfaces for times ranging from three hours to nine days [5]. The COVID-19 virus spreads via contact and the respiratory system, with coughing and sneezing by an infected individual being the primary sources of respiratory droplets [6]. The WHO recommended wearing a mask to prevent the spread of disease in public. The face mask market was worth US$0.79 billion in 2019 and reached around US$166 billion in 2020 [7]. PPE kits and N-95 masks were not produced in India till January 2020; instead, 2.75 lakhs of them were imported. Yet by the end of May 2020, India became the second-largest producer of PPE kits, churning out almost 4.5 lakh PPE kits per day and more than 104 lakh N-95 masks [4].

### Bio-Medical waste

One of the more prevalent pollutants produced by healthcare institutions is bio-medical waste (BMW), which is produced during the medical diagnosis, treatment, or immunization of people, animals, and biological research activities [8]. Biomedical waste is defined as any waste, which is generated during the diagnosis, treatment, or immunization of human beings or animals, or in research activities pertaining thereto, or in the production or testing of biologicals," states the Biomedical waste (Management and Handling) Rules, 1998 of India [8]. In the context of COVID-19, biomedical waste includes waste produced during patient treatment, diagnostic, isolation, and at-home care. Solid waste is only recognized infectious if it has been contaminated by COVID-19 patients' body fluid and secretions (such as tissues, masks, gloves, etc.); otherwise, uncontaminated solid waste is managed in accordance with the Solid Waste Management Rules, 2016 [9]. According to guidelines, leftover food from infected persons, used water bottles, food plates, surgical masks, gloves, bibs, rolls, sheets, wrappers of medications and syringes, and semisolid gels are included under the purview of BMW [10].

### Classification of Bio-Medical waste

**Figure 1.**
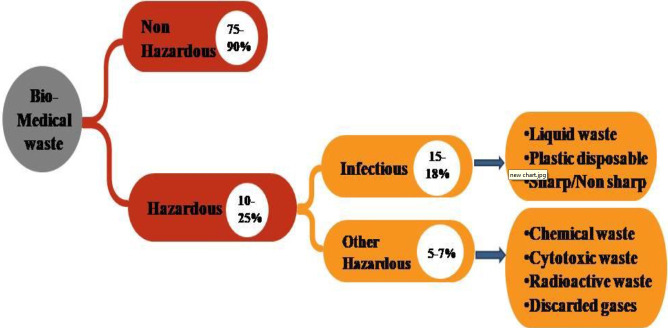
Classification of Bio-Medical waste [12]

Bio-Medical waste can be classified as non-hazardous and hazardous waste as depicted in Figure 1.

(a) Non-hazardous waste: In a healthcare facility, nonhazardous waste makes up around 75 to 90% of the waste. This waste covers elements like leftover food, paper cartons, fruit peels, packing materials, etc. It doesn't cause any special biological, chemical, radioactive, or physical risks [11].

(b) Hazardous waste: The remaining 10–25% of medical waste is hazardous, which means it could harm people, animals, or the environment [11].

## Methodology

This paper primarily reviews the types, amounts, and impacts of medical waste produced during the COVID-19 epidemic and management strategies and treatment technologies of biomedical waste. Bio-Medical waste, COVID-19, Waste generation, Type of waste, Impacts, Corona virus were some of keywords used in the desktop search for the papers. Scopus, Web of Science, Research Gate, Science Direct and Pubmed were the five journal databases used in the search. The review considered academic works, including peer-reviewed journal articles and conference papers that were written in English. Also, publications from several non-academic sources (such news websites, etc.) and up-to-date guidelines, including those from the Ministry of Health and Family Welfare, Government of India, were accessed. To narrow the search, the terms were also combined such as COVID-19 and waste management. The search turned up 189 articles, which were then checked for applicability to the review. The following requirements must be met in order for an article to be considered for acceptance:

1) It must be about medical or healthcare waste, not other types of waste.

2) The waste must have been produced during the COVID-19 pandemic.

3) It must include information about the quantity, variety, and types of medical or healthcare waste that was produced.

4) It must discuss the waste's effects.

5) It must have management and treatment strategies for the biomedical waste.

**Figure 2.**
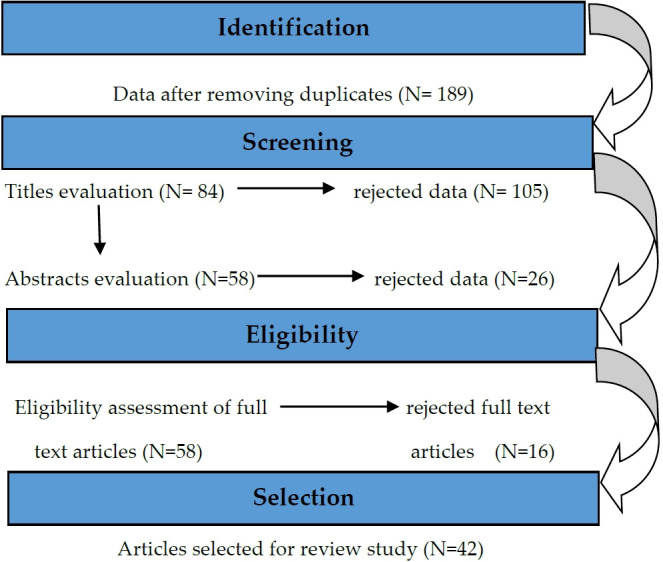
Methodology adopted for the review study.

All articles pertaining to COVID-19 and waste creation were chosen in the initial step. Articles that did not meet the aforementioned requirements were disqualified. The articles' headings and summaries were assessed during the second screening. The previously chosen articles were assessed in the third and final screening process. This led to the finalization of 42 publications for the study. (Figure 2).

## Results and Discussion

**Figure 3.**
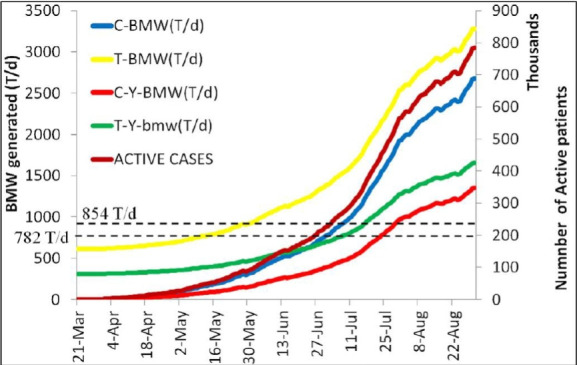
Different forms of BMW produced in India every day during COVID-19 [13]

India's already-stressed waste disposal system has been strained by the COVID-19 outbreak. The extremely contagious nature of COVID-19 has resulted in an unprecedented demand for PPE kits and other single-use medical equipment, which has caused a tremendous generation of the yellow category of biomedical waste (Y-BMW). The yellow category of Bio-Medical waste comprised of Human/Animal anatomical waste, Soiled waste, expired or discarded medicines, chemical waste, chemical liquid waste, laboratory waste and mattresses or beddings contaminated with blood or body fluids [13]. During the pandemic, it was predicted that each person's daily disposal of a single-use face mask may produce 66 kT of Y-BMW in a year [13]. The extensive use of medical technology in hospitals and safety precautions to prevent the spread of the disease has caused a significant increase in BMW production [14]. In 2017, India created a total of 557 tonnes of BMW each day, of which 517 tonnes per day were left untreated, according to the central pollution control board (CPCB, India) [15]. India has 198 certified BMW treatment facilities, and another 28 were being built [16]. These CBWTFs (Common Bio-Medical Waste Treatment Facilities) managed 710T/d of the BMW through May 2020, 101T/d of which were from COVID-infected persons [13]. According to the CPCB's 2019 annual report, 233 incinerators are operational across several Indian states. The daily generation of different forms of BMW from March to August 2020 in India during the pandemic, is depicted by Figure 3. It is evident that after May 30, 2020, the number of COVID-infected patients increased rapidly. The generation of T-Y-BMW also grew under these conditions, and by the 13th of July 2020 and the 10th of July 2020, it was noted that the incineration capacity of India's CBWTFs, while including captive incineration and excluding captive incineration, respectively, was fully employed. The incineration capacity of India's CBWTFs was shown by two horizontal dotted lines, one with captive incineration (854T/d) and the other excluding captive incineration (782T/d) [13]. On July 27, 2020, the quantity of C-Y-BMW (COVID-Yellow BMW) alone was able to surpass India's Y-BMW incineration capacity. Moreover, it can be seen that in the event of a pandemic, the rate of C-Y-BMW production remained steady until August 31, 2020 [13].

**Figure 4.**
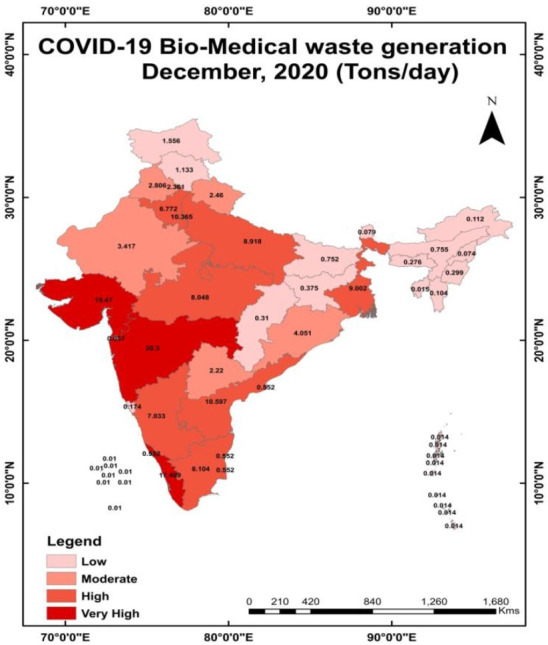
State wise COVID-19 BMW generation in December, 2020 (Tons/day) [19]. Map created by the author.

The sudden increase in PPEs, other medical equipment, etc. as a result of the COVID-19 outbreak has negatively impacted the waste management system. BMW increased 850 tonnes per day in India in 2020 as a result of the pandemic situation. [15]. In 2021, India had a 46% gross increase in the number of BMWs nationwide [15]. The production of COVID-19 waste was spread throughout 2,907 hospitals, 20,707 isolation facilities, 1539 collection sites, and 264 testing facilities [16]. Maharashtra, Kerala, Gujarat, Andhra Pradesh and Delhi were top five states/UTs which generated COVID-19 related BMW during December 2020 as shown in Figure 4. In Delhi, cotton swabs used in the reverse-transcription polymerase chain reaction (RT-PCR) assays for COVID-19 are most likely the source of the greater biomedical waste generation rate [17]. The waste generation rate has increased dramatically as a result of COVID-19, reaching 3.4 kg/person/day. The private emergency clinics in Delhi were found to generate medical waste at a rate of 0.5 kg to 4 kg per bed each day [18].

### Types of Bio-Medical waste during COVID-19

**Figure 5.**
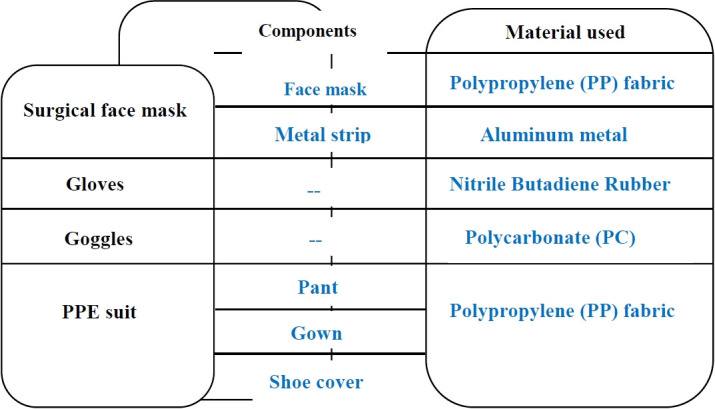
PPEs categories and their composition [25]

Human COVID-19 transmission could be reduced by following several guidelines, including keeping a distance from others, putting face masks or shields, regularly washing your hands, and using hand sanitizer. Face masks, aprons, rubber boots, shoe covers, gloves, face shields, medical test kits, and plastic containers included the essential PPE for medical staff caring for patients with COVID-19 [20]. Throughout COVID-19, masks have been extensively utilized as a key defence against the spread of the SARS-CoV-2 virus that is responsible for COVID-19, while PPE including gloves and coverall suits were mostly used by medical staff [18]. Moreover, using COVID-19 testing apparatus generated a lot of medical waste. For COVID-19, mouth and nasal swabs have been utilized consistently during screening and other needed testing [18]. 89 million medical masks, 76 million gloves, and 1.6 million medical goggles were expected to be needed each month of this pandemic, according to WHO modelling [21]. With regard to the COVID-19 control policies, the type and quantity of medical waste associated to COVID-19 varies throughout time and place [18]. A variety of plastics, including polyethylene (PE), polyethylene terephthalate (PET), high-, low-, and linear-low-density polyethylene (HDPE, LDPE, and LLDPE), polyvinyl chloride (PVC), polypropylene (PP), and polystyrene, are used in the production of PPEs [21]. The used coveralls are constructed of non-woven material, is actually made of polypropylene. Non-woven fabrics using PP and PE are used to make face masks. These nonwoven synthetic textiles have degraded, creating microplastic and microfibers. These polymers break down into tiny microplastic fragments. In 2020, 1.56 billion face masks were estimated to have entered the oceans [22]. Face masks are confirmed in recent studies to be possible causes of microplastic pollution in water systems [22]. While cleaning, disinfecting, or providing care and/or treatment for COVID-19 patients, gloves are important and necessary. Medical personnel must wear two or three layers of surgical gloves when entering high-risk areas to protect themselves from infection [23]. When using various tools that aid in diagnosis and treatment, it is crucial to use goggles and protective face shields or screens to safeguard those who might be exposed to blood or other potentially infectious fluids or viruses. Moreover, using COVID-19 testing apparatus generates a lot of medical waste. For COVID-19, mouth and nasal swabs have been utilized consistently during screening, routine, mass, and other needed testing. Swabs and specimen holders that have been used are labeled as biohazards and transferred to medical incinerators for burning [24]. Figure 5 illustrates PPEs categories and their composition (Personal Protective Equipments).

### Management of Biomedical waste in India

The management of biomedical wastes has grown to be a significant issue in India as well. Concerning the management of biomedical wastes linked to environmental health, there is an urgent demand for the planning and implementation of updated methods and practices at different planning levels [26]. For effective management, some important steps such as collection, segregation, storage, transportation, treatment and final disposal are required [27]. The various goals of BMW management are [28]:

• To stop the spread of pathogens and diseases

• To stop environmental damage.

• To avoid widespread exposure to the cytotoxic, genotoxic, and chemical impacts of biomedical waste.

• To avoid harming healthcare professionals and those who handle BMW.

The Central Pollution Control Board (CPCB) of India has issued rules on the disposal of waste created during the treatment, diagnosis, and quarantine of COVID-19 patients in response to the COVID-19 outbreak in India [29]. In order to ensure that COVID-19 waste is systematically collected and delivered to the "Biomedical Waste Disposal and Disposal Facility," the CPCB periodically assessed the needs of the Bio-Medical Waste Management (Amendment) Rules 2018 [29]. Health Care facilities (HCFs) are largely responsible for managing the healthcare waste produced inside of the facility, including any community-based work they do. Prior to being collected by the Common Bio-medical waste Treatment Facility (CBWTF) Operator, the health care facilities are responsible for segregating, collecting, internal transportation, pre-treating, and storing the waste that they generate. Therefore, to ensure efficient waste management in healthcare facilities, every group of personnel must comprehend and put into practice the technical requirements of waste handling by the BMWM Rules, 2016. Based on the segregation pathway and color code, the Bio Medical Waste Management Rules, 2016, divide the bio-medical waste produced by the healthcare facility into four categories [30]. Each of the categories is further assigned different kinds of biomedical waste, as explained in the Table 1.

A smartphone application called COVID-19 Biomedical Waste Management, which was launched by CPCB in May 2020, allows users to track rapidly expanding waste flow in real-time [29]. The CPCB has adopted the additional steps listed below to improve the management of biomedical waste during the COVID-19 epidemic [19].

• As per Biomedical Waste Management Rules 2016, different color coded bins, bags or containers were kept in wards to maintain segregation of waste.

• To avoid bag leakage, waste from the COVID-19 isolation ward was collected in a two-layer bag (Indian Council of Medical Research 2020).

• The biowaste from the COVID-19 isolation ward was documented separately.

• Bins and trolleys used for COVID-19 trash should be properly disinfected each day.

• Urban Local Bodies (ULBs) provided yellow-colored bags that were used to collect waste products from COVID-19 quarantine centers.

• Separately assigned specialized sanitation personnel to handle biomedical waste and general solid garbage so that waste can be collected and transported to a temporary disposal site on time.

• Used Personal Protective Equipments (PPEs) such as gloves, plastic coveralls, face shields, splash-proof aprons, and goggles were collected into a red bag.

• Used masks, caps, shoe covers, and disposable linen gown and non- or semi-plastic coveralls were collected in yellow bags.

• At the point of generation, in the wards or isolation rooms, biomedical waste and regular solid waste should be separated.

• Masks and gloves that were used by anybody other than COVID-19 patients should be kept in a paper bag for at least 72 hours after being cut to prevent reuse.

• To update the information on the development of COVID-19 biomedical waste, register for the CPCB mobile application called "COVID19BWM."

### Treatment methods and disposal of Biomedical waste

Treatment of biomedical waste is the process used to get rid of the waste's negative consequences. Numerous treatment techniques are available to ensure the highest level of safety during waste management and disposal. Additionally, it lessens environmental risks [30]. The techniques used are autoclaving, thermal inactivation, incineration, gas sterilization and chemical disinfection, microwave irradiation and mechanical processes such as compaction, shredding. [3,28,32].

#### 1) Autoclaving

Steam is brought into direct contact with waste in a regulated manner for long enough to disinfect it via the lowheat thermal procedure known as autoclaving [33]. Gravity flow autoclaves and vacuum autoclaves are the two types of autoclaves that are used to disinfect the waste [28]. Low-density materials like plastic, metal, bottles, and flasks are best for steam sterilization since they are less dense [12]. High-density polyethylene and polypropylene plastics shouldn't be used in this procedure since they don't allow steam to penetrate the waste load. To minimize spills and drain clogs, plastic bags should be placed in a hard container before steam treatment [12]. In hospitals, it is one of the most widely used methods of treating the waste. Autoclaves can handle up to 90% of medical waste [32].

#### 2) Incineration

Basically, it is a burning technology. It is a controlled combustion process where waste is fully oxidized and any harmful microbes are killed or denatured at high temperatures [28]. By converting organic combustible waste into inorganic incombustible waste, this reduces the volume and weight of the wastes dumped in landfills [32]. The incineration setup requires significant capital and operating costs for new technologies. For this method, the waste should be combustible and its moisture content should be less than 30 percent [12]. The main drawback is that incinerators produce pollutants like carcinogenic dioxins and furans, heavy metals, particulates and toxic gases [34].

#### 3) Thermal inactivation

In this method, the waste is exposed to high temperatures for longer time duration to remove infectious pathogenic organisms. It is used to treat a large volume of biomedical waste. The temperature and duration of the treatment are dependent on the types of pathogens in the waste [12]. Waste liquid is collected in a container and heated using heat exchangers or a steam jacket surrounding the container [32]. Following treatment, the contents may be disposed of in accordance with local or state regulations into the municipal sewage [12].

#### 4) Gas sterilization

Chemicals that are gaseous or evaporated are used in gas/vapor sterilization as the sterilizing agents. The most widely utilized substance is ethylene oxide, however since it may cause cancer in humans; it should be used with caution. When handling sterilized items, there is a chance that workers could be exposed to ethylene oxide since it can adsorb on the surface of treated materials [12].

#### 5) Chemical disinfection

For liquid pathogenic wastes such as blood, urine, stool etc. the process of chemical disinfection is the preferred method of treatment [32]. Chemicals are used to disinfect the waste. Examples of such chemicals include ozone, hydrogen peroxide, peracetic acid, sodium hypochlorite, dissolved chlorine dioxide, and dry inorganic chemicals. The majority of chemical reactions require neutralizing agents and a lot of water [28].

#### 6) Microwave irradiation

In this process, microbes are destroyed by the heat created by electromagnet beams. The majority of the microorganisms are killed at a frequency of around 2450 MHz, which varies from 300 to 300,000 MHz. this method is not suitable for cytotoxic, hazardous and radioactive waste [32].

#### 7) Mechanical processes

Shredding and compaction are two important mechanical methods to alter the physical qualities or shape of waste for easier management of the waste. Compaction is a technique for reducing the volume of garbage. Paper and plastic garbage are destroyed through shredding to avoid their reuse. A shredder can only be used with waste that has been disinfected [28].

### Waste disposal after treatment

The infectious waste is transformed into non hazardous waste only after treatment. After the treatment, the waste is no longer biologically hazardous and can be mixed with general solid waste for final disposal [12]. The enormous quantity of BMW has been successfully disposed of using land disposal or burial. At the same time, a landfill is not an ideal method to dispose of biomedical waste because of its negative impacts on the environment and human health [35]. These negative impacts include groundwater and soil pollution through leachate formation and various gas emissions during the process of degradation of waste in a landfill. To ensure the safe disposal of MW, preventive steps should be carried out including [35]:

• High frequency of covering the waste,

• Burying the waste for a minimum of three months under old municipal solid waste,

• Water-resistant bottom

• Waste layers should be at least 2 meters above the water table,

• No chemical dumping.

### Impacts of COVID-19 related Bio-Medical waste on different environmental components and human health

**Figure 6.**
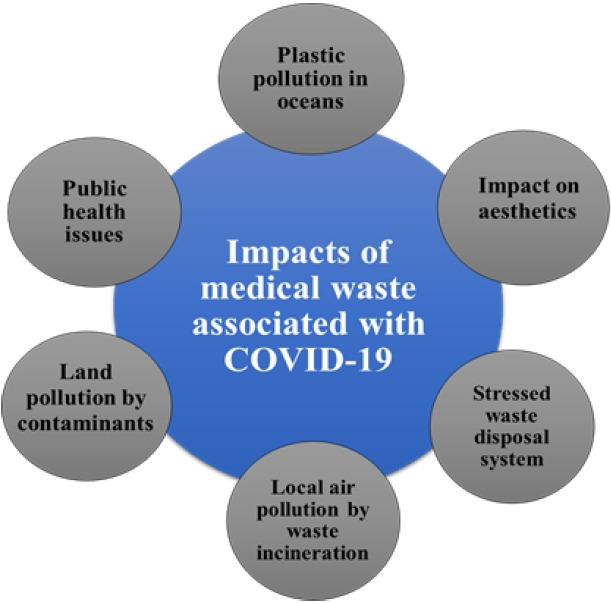
Major impacts of COVID-19 Bio- Medical waste generation.

As is well known, developing nations like India lack the necessary technological advancements to effectively manage any foreseeable waste disaster [21]. Human health and safety always take precedence over other concerns during pandemics. The management of medical waste was one of the pandemic's most significant repercussions. Fears about the corona virus spreading through medical waste prompted the development of separate systems for collecting, storing, and transporting this potentially infected waste [3]. COVID-19 related waste had a significant impact on environmental factors like soil, water, and air. The environmental footprint has undergone a significant alteration as a result of the epidemic. Approximately, for every 1000 corona virus tests, PCR (Polymerase Chain Reaction) procedures produce about 22 kg of plastic waste [20]. Reverse transcription PCR (RTPCR) is known to produce 37.27 g of plastic remnants per sample [20]. The major impacts of Bio-medical waste generation during COVID-19 are shown in Figure 6.

### Impact on water quality

Hospitals, laboratories, isolation facilities, quarantine wards, homes, public markets, businesses, and workplaces are just a few of the areas where wastewater is produced that could spread the COVID-19 virus. Wastewater must be properly treated as a preventative step since polluted wastewater could serve as a possible source of transmission. Studies have revealed the existence of SARS-CoV-2 strains in infected people's faeces, which could then enter wastewater. The COVID-19 virus was found in the stool samples of infected people, indicating that faeces served as the route of transmission [21]. The amount of plastic in the aquatic environment is growing since some of PPEs (such as face masks, gloves etc.) used to prevent the disease are making their way into streams and eventually ending up in freshwater and the ocean and ultimately affecting marine ecosystems [36]. Marine species at different trophic levels have been reported to have microplastics that have penetrated the food chain. They have thus infected human meals [18]. Groundwater sources could become contaminated as a result of improper biomedical waste segregation and disposal, which could then infect both people and animals [6]. In some cases, water bodies close to incineration operations have been found to contain dioxins . Dioxins enter the water body through the air. [37].

### Impact on land or soil quality

Improper and unscientifically COVID-19 Bio-Medical waste disposal at dumping sites may contaminate the soil nearby and may result in subsequent transmission of the disease. The chemistry and biology of the soil ecosystem may change as a result of various contaminants interacting with the soil [38]. If non-biodegradable and non-recyclable biomedical waste is dumped on land without following the required decontamination procedures, it could linger there for years and be carried by wind or water movement [23].

### Impact on air quality

The lockdown imposed due to the pandemic had an impact on indoor air quality. Indoor air quality got worsened because all people had to stay at home during lockdown periods. The number of indoor air pollutants (such as black carbon from smoke) was high. Several disinfectants, such as isopropyl alcohol, chlorine derivatives (liquid chlorine, sodium hypochlorite, and chlorine dioxide), and ozone were employed to sanitize environmental surfaces (both animate and inanimate), medical waste, and wastewater [39]. Such disinfectants' fumes have the potential to significantly affect both air chemistry and human health. NaOCl droplets combine with water vapor to create HOCl, which then undergoes photodissociation to produce Cl radical. In order to alter the atmospheric chemistry, the Cl radical causes secondary aerosol creation, tropospheric ozone removal, and sulfate aerosol production [21].

Incineration of Bio-Medical waste could harm the environment and mix various chemicals to harmful levels for human health. During incineration, used PPEs and SUP (single-use plastic) products in C-BMW are likely to produce additional dioxins, furans, PAHs, and polychlorinated biphenyls (PCBs), which are highly dangerous organic chemicals [14]. Studies have indicated that burning plastics produces more unintended persistent organic pollutants such as PCBs, PAHs, dioxins, and furans [14]. These harmful gases and by-products can harm the immune system and result in reproductive and developmental issues as well as serious illnesses like cancer [40].

### Impact on human health

Pathogens, genotoxicity, radioactivity, the inclusion of hazardous compounds, the presence of sharps, needles, and hazardous chemicals all contribute to the hazardous nature of bio-medical waste [41]. The transmission of illnesses is facilitated by improper behaviors such as the disposal of biomedical waste in public dustbins, open areas, water bodies, etc. COVID-19 Bio-medical waste posed a threat to the following categories of people: Medical professionals, including doctors, nurses, sanitary staff, and hospital maintenance staff; Patients receiving care in healthcare facilities, both inpatients and outpatients, as well as their visitors [11]. The microorganisms present in COVID-19 related waste enter the body by inhalation, assimilation, mucus, wound/cut, and skin abrasion [41]. Infectious (masks, gloves, PPE kits), pathological, pharmacological, chemical, and radioactive natures are all represented in the Covid-19 biomedical waste. Although the duration of the virus's survival on surfaces is unknown, according to various studies, it could last anywhere from a few hours to a few days. This can also be caused by changes in temperature or humidity in the surroundings [42]. According to an estimate, 5.2 million individuals worldwide die each year from illnesses brought on by medical waste [43]. On the other side, open burning and incinerator emissions expose people to risky chemicals that might cause cancer and respiratory conditions [37]. Generally, the various diseases caused by hospital waste in humans are presented in Table 2 [45].

### Cost effective and eco-friendly biomedical waste management methods

The techniques used for the treatment and disposal of biomedical waste carry the risk of public health issues, environmental pollution, and occupational challenges. The process of incineration emits dioxins and furans, which cause neurological disorders in children, reproductive issues in women, and the threat of skin cancer in public [46]. The ash left behind after incineration is also dangerous; therefore, it is necessary to measure the toxins present in the ash before disposing of it in a landfill [47]. Chemical-based methods use various chemicals to disinfect wastes like sharps, laboratory waste, cultures, etc., which are also harmful [47]. Microwaving and hydroclaving don't appear practical in the context of countries with limited resources [46]. Leachate formation and gas emissions are major problems due to waste decomposition in a landfill [47]. To avoid these impacts, most countries are switching to environmentally friendly and cost-effective techniques to dispose of biomedical waste. These techniques may help to mitigate the adverse impacts of waste on human health and the environment by inhibiting harmful substances [48]. However, it also encounters certain limits and difficulties while applying biotechnology in waste management, like moral, social, legal, and economic concerns. Some environment-safe methods to manage biomedical waste are discussed here.

#### Bioremediation:

It is the process of using living organisms or their by-products to treat or degrade toxins into less toxic or nontoxic substances that are present in the environment [49]. The enzymes that are genetically modified and have high activity, known as recombinant enzymes, may be utilized to treat medical waste [48]. One of the most promising examples of such an enzyme is Horseradish peroxidase (HRP), which can be used to degrade medical waste. Various organic pollutants like phenols and polycyclic aromatic hydrocarbons (PAHs) are usually identified in medical waste and can be easily catalyzed by HRP comprising the heme group [48]. Another example of a recombinant enzyme that can be used in the bioremediation of medical waste is Laccase. Laccase, an enzyme containing copper, can promote the oxidation of various organic compounds, including lignin, a complex polymer found in plant matter and a significant proportion of some medical waste streams [48]. A study reveals that the saprophytic, Coprophilic fungus Periconiella sp. present in Desi Cow’s dung has been found a good decomposer of biomedical waste like dressings, soiled pieces of cotton, surgically removed material etc. within around 40 days [46]. Other examples of such useful enzymes include lipases, proteases, and cellulases which can degrade lipids, proteins, and celluloses, respectively [48].

#### Phytoremediation:

It is a type of bioremediation technique in which plants and different types of microorganisms that shelter on plants are used to remove contaminants from the environment [48]. It may prove cost-effective and long-lasting alternate option for eliminating medical waste. Vetiver grass (Chrysopogon zizanioides) has been found very promising at eliminating various diseases causing organisms, organic pollutants, and different heavy metals present in biomedical waste [48]. Likewise, a combination of tobacco and neem extracts was found three times more cost-effective and superior to techniques currently used to treat medical waste [47]. Willow (Salix spp.) has been proved very effective in treatment of infected medical waste. It has shown its potential to purify contaminated soil, water, and air [50]. It shows high sensitivity to contaminants and is capable to store them in its own tissues. Additionally, it may enhance some beneficial soil bacteria which can help to decontaminate the waste [48].

#### Composting:

A biodegradable component of biomedical waste can be treated naturally and sustainably by the means of composting. It is a biological method that can be adapted to recycle organic materials and the final product is a potential soil fertilizer or conditioner with high nutrient level [47]. This method can be applied to a variety of items of medical waste, such as leftover food from hospitals or clinics, discarded or expired medications, and other organic waste materials [48]. Various types of microorganisms like bacteria, fungi, and actinomycetes degrade complex structured organic material to simple structured substances comprising organic acids, carbon dioxide, and water. Bacillus subtilis and Aspergillus niger are some examples of microorganisms frequently used in composting [48]. Cellulose, lipase, and protease are recombinant enzymes that can be utilized to manage medical waste by composting. Medical waste such as bandages, cotton swabs, soiled linen, surgical drapes, and body fluids such as blood, etc. can be treated with these enzymes. These enzymes can fasten the rate of decomposition of complex structures of organic materials, help in volume reduction of waste, and ultimately give a final product as compost, which is nutrient-rich and used as a soil conditioner [48].

#### Vermicomposting:

Vermicomposting is also supposed to sterilize infected medical waste components without harming the environment or public health. A performance study by Dinesh et al. (2010) was done using different species of earthworms separately and as a mixed culture to degrade biomedical waste. Eisenia fetida, Eudrilus eugeniae, Perionyx excavatus and mixed culture of these worms were employed for decomposition of waste [50]. Initially, the collected medical waste was treated with cow dung slurry to degrade the waste at the primary level after sterilizing the waste with NaOCl. This decomposed medical waste was offered to the earthworms in monoculture and polyculture practices. In this study, E. Fetida was more effective than the two epigeic earthworm species, P. excavatus and E. eugeniae. The mixed culture of these three species of worms was also found equally effective as E. Fetida in decomposing medical waste [50]. Thus vermicomposting can also be used as an alternative method for treatment of biomedical waste.

## Conclusions

Most countries generate a significant amount of medical waste, of which approximately 75% is non-hazardous. The rest of it is regarded as dangerous because it is infected with pathogens that can spread numerous diseases and infect people, thus medical waste must be handled and treated with care. The most significant effects of COVID-19 on waste management in India can be summed up as a boost in household waste, an upsurge in the amount of plastic waste generation, a decline in littered waste versus a rise in the risk to the environment and health of this waste type, an abrupt rise in the generation of medical waste, and the generation of infectious substances outside of hospitals (homes with sick or self-quarantined people). The threat of improper and open dumping of toxic biomedical waste must be stopped, and this can only be done by the government with constant oversight and dedicated regulatory authorities. Generally, minimization of bio medical waste at the place of its origin is the key step towards its management. As we can observe in our surroundings the improper handling of biomedical waste is caused by a lack of knowledge and awareness. It is necessary to raise awareness among the general public about BMW and the significance of keeping it separate from other waste.

## Figures and Tables

**Table 1.** Puncture proof, leak proof boxes or containers with blue colored marking.

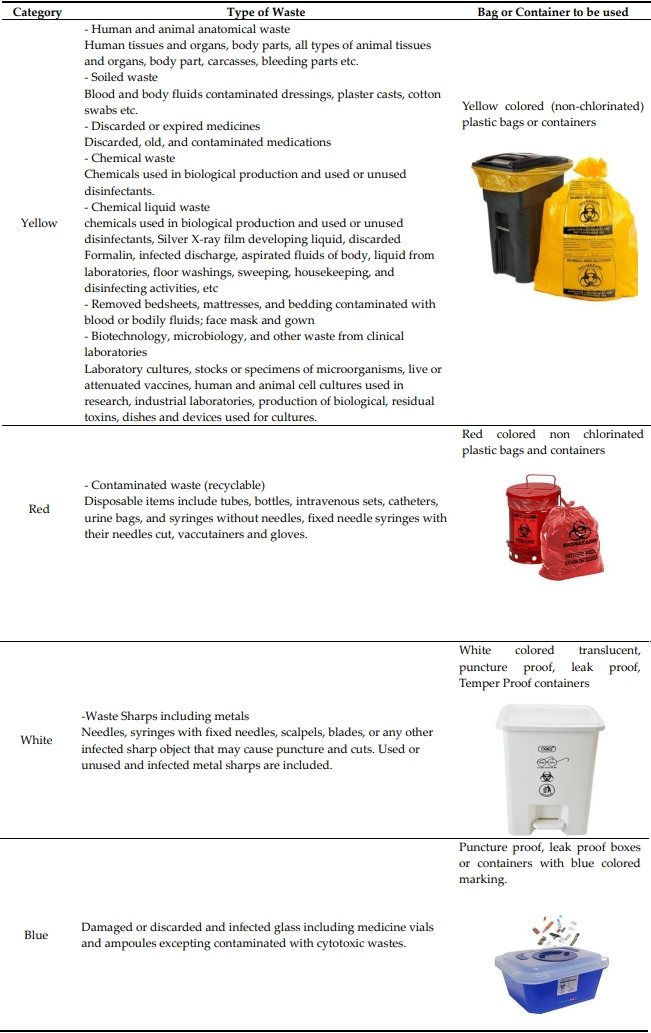

**Table 2.** Various diseases caused by BMW in humans

Disease causing organism	Disorder caused	Example of hospital waste
**Viruses**	AIDS, Infectious Hepatitis, Dengue, tick-borne fevers, Japanese encephalitis etc.	Biological fluids, stained linen, excreta of human, contaminated needles.
Hepatitis A,B,C, HIV, Enteroviruses, Arboviruses		
**Bacteria**	Cholera, Tetanus, Typhoid, wound infections, rheumatic fever, endocarditis, infections of skin and soft tissues in body.	Excreta of human, biological fluids, sharp objects like needles, surgical knives in hospital waste.
Vibrio cholera, Salmonella typhi, Pseudomonas, Clostridium Tetani, Streptococcus		
**Parasites**	Malaria, Kala azar, Cutaneous lesih maniasis	Blood and biological fluids, Excreta of human.
Plasmodium, Wucheraria Bancrofti		
